# Are New Yorkers Losing Their Eyesight?

**Published:** 1890-05

**Authors:** S. H. Preston


### ARE NEW YORKERS LOSING THEIR EYESIGHT?

By S. H. Preston.

A startling speculation, but one suggested to an observant visitor; everywhere victims of defective vision; scarcely a person seen in street cars reading a paper with unaided eyes; school children, and those in charge of nurse maids, wearing spectacles or eye-glasses.

Take notice of this matter. Inquire of oculists and at ophthalmic hospitals. The trouble is increasing at an alarming rate ; scores of causes are assigned ; tea, tobacco, adulterated food, overtaxed nerves, too much work or worry, and too little sleep, etc.

But stand out here on this corner and look up where the sunshine shimmers across the avenue. See the shower of sparkling dust rising from that sanded car track, it is like a fine fog, filling all the air ; it also fills the eyes and lungs of the passers-by.

And now overhead rumbles and roars and rattles by, the ponderous elevated trains, grinding the stilted steel track, and shaking it from one station to another. All along its course a shower of soot and cinders and steel dust sifts down upon the street.

The microscopic sand and metallic mist from the street are met and whirled back by the smoke and dust clouds from above; the atmosphere is permeated with powdered stone and steel. The eyes feel filmy, and itch and smart ; the majority one meets are rubbing theirs with handkerchiefs. And this is the state of things all along the miles and miles of car tracks throughout the city every second of the day. Go into any of the houses along the elevated lines ; however tightly the front windows and shutters are closed, the inside ceilings and curtains are covered with a dark dust ; the bowl upon the wash-stand, which was wiped clean an hour ago, will be crusted over again. These miles of stone and sand and steel are being ground and rubbed to powder by ever-rolling wheels, and whirled into our eyes.

And this makes a demand upon inventive genius. What is wanted, is a veil or visor, of finely woven wire, magnetized to catch these floating metallic atoms—a scientific shield against this sight-destroying dust. Fame, fortune and a monument should be awarded the designer of such a magnetic mask.

May be man's physical faculties of sense are destined to die out. That animal sense of scent that enables the deer to detect the proximity of a foe, the dog to follow the trail of his master, has become entirely extinct in man. Defective sight, hearing, etc., increase with the amenities of modern life. American aborigines had no need of spectacles or ear-trumpets, oculists or dentists. The old rudimental senses are becoming dull, and may be are destined to disappear through disuse as man grows from grosser to finer grades of being, and develops more fitting and perfect faculties. The many marvellous manifestations of catalepsy and somnambulism, with phenomenal instances of persons, like the late Laura Bridgeman, who seem to have acquired strange new senses to compensate for those lost, make it conceivable that mankind may, in some far off millennial future, come into a clairvoyant and clairaudient condition, in which eyes and ears will be of no further use.

